# Biolayer interferometry predicts ELISA performance of monoclonal antibody pairs for *Plasmodium falciparum* histidine-rich protein 2

**DOI:** 10.1016/j.ab.2017.07.010

**Published:** 2017-10-01

**Authors:** C.F. Markwalter, I.K. Jang, R.A. Burton, G.J. Domingo, D.W. Wright

**Affiliations:** a Department of Chemistry, Vanderbilt University, Nashville, TN 37235, USA; b PATH, Seattle, WA 98121, USA

**Keywords:** Biolayer interferometry, ELISA, Malaria, Histidine-rich protein 2, Binding kinetics

## Abstract

Predicting antibody pair performance in a sandwich format streamlines development of antibody-based diagnostics and laboratory research tools, such as enzyme-linked immunosorbent assays (ELISAs) and lateral flow immunoassays (LFAs). We have evaluated panels of monoclonal antibodies against the malarial parasite biomarker *Plasmodium falciparum* histidine rich protein 2 (HRP2), including 9 new monoclonal antibodies, using biolayer interferometry (BLI) and screened antibody pairs in a checkerboard ELISA. This study showed BLI predicts antibody pair ELISA performance for HRP2. Pairs that included capture antibodies with low off-rate constants and detection antibodies with high on-rate constants performed best in an ELISA format.

## Highlights

• Kinetic parameters of 15 anti-HRP2 antibodies are measured by biolayer interferometry.
• Kinetic constants are compared to a checkerboard ELISA of 225 antibody pairs.
• Biolayer interferometry predicts antibody pair performance for HRP2 ELISA.
• Capture mAbs with low *k*_*off*_ and detection mAbs with high *k*_*on*_ are best in HRP2 ELISA.

## Graphical abstract

**Figure:**
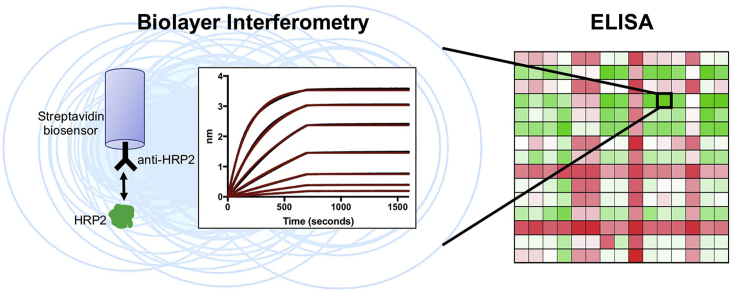

Antibody-based diagnostics for disease-specific biomarkers are invaluable tools for patient care, disease surveillance, and intervention management. Lateral flow immunoassays (LFAs), which leverage high-affinity and specific antibody-antigen interactions in a sandwich immunoassay format, make infectious disease diagnosis possible in low-resource settings, where laboratory infrastructure is unavailable. For example, approximately 314 million antibody-based LFA rapid diagnostic tests for malaria were sold in 2014. Most of these tests were deployed in sub-Saharan Africa, where the disease burden is high and good-quality, high-throughput microscopy is not readily available [1].

While LFAs have made malaria diagnosis accessible and affordable, they lack the sensitivity needed to detect low parasite densities. Submicroscopic and asymptomatic cases are often missed by these tests and left untreated, remaining a reservoir for malaria transmission [2], [3], [4], [5]. Recent models suggest that diagnostics with detection limits of 200 parasites/μl only detect 55% of the infectious reservoir, depending on the setting [6]. These data highlight the need not only for more sensitive malaria rapid diagnostic tests, but also for laboratory tools with limits of detection capable of defining the clinically relevant protein biomarker concentrations required to accurately diagnose asymptomatic infections.

The primary protein biomarker used in malaria rapid diagnostic tests for *Plasmodium falciparum* infection is *Plasmodium falciparum* histidine-rich protein 2 (HRP2), which is expressed by one out of five species of malaria parasites known to infect humans. HRP2 is a unique biomarker because it lacks native tertiary structure, and its sequence is 30% histidine, consisting largely of AHHAHHAAD and AHHAAD repeat motifs [7]. Expression of HRP2 varies over the erythrocytic life cycle of the parasite, though the function of the protein remains unconfirmed [8], [9].

Sensitive antibody-based diagnostics and laboratory tools for HRP2 detection at extremely low parasite densities will depend on high-affinity molecular recognition events for both capture and detection of the biomarker. Biolayer interferometry (BLI) is a label-free bioanalytical technique used to quantify the strength of antibody-antigen interactions, allowing for measurement of kinetic parameters such as the dissociation constant (*K*_*D*_), on-rate constant (*k*_*on*_), and off-rate constant (*k*_*off*_) [10], [11]. This optical technique allows for real-time monitoring of the interference pattern of white light reflected from two surfaces within fiber optic sensors that are immersed in biomolecule solutions. This experimental set-up is advantageous over evanescent (eg: surface plasmon resonance) or acoustic label-free systems, which typically require microfluidics to deliver the sample to the sensing surface. These systems are prone to clogging when complex sample matrices are used. Further, since BLI detection occurs at the biosensor tip surface, matrix effects, such as those from unbound proteins in solution, are minimized [11].

The crucial need for improved malaria point-of-care diagnostics and laboratory assays and the reliance of these tools on the strength of antibody-antigen interactions highlight the importance of selecting the best monoclonal antibodies (mAbs) for capture and detection. The goal of this work is to assess biolayer interferometry as a tool for predicting antibody pair performance in an enzyme-linked immunosorbent assay (ELISA) for HRP2. The kinetic parameters of 9 novel mAbs as well as 6 commercially available clones (Table S1) are determined and compared to antibody pair performance in a sandwich ELISA format.

The strength of mAb-rcHRP2 interactions was measured using BLI. For each anti-HRP2 IgG mAb, the antibody was biotinylated, loaded onto streptavidin biosensors, and HRP2 was allowed to associate and dissociate. Specific experimental conditions can be found in Supplemental Information. The binding profiles, pseudo-first order fit curves, and residuals are plotted in Tables S2 and S3. Kinetic parameters for all anti-HRP2 mAbs are listed in Table S4. Notably, 11 out of 15 anti-HRP2 antibodies had off-rate constants below the limit of detection of the OctetRed96 instrument (<1 × 10^−7^ 1/s). Although experiments were optimized such that one-to-one fits could provide accurate estimations of mAb-HRP2 affinities, it is likely that repeated motifs throughout the protein allowed for re-binding of HRP2 to the mAb-functionalized sensors during the dissociation phase, resulting in no net dissociation.

While the off-rate constants make distinguishing the anti-HRP2 mAbs by *k*_*off*_ or *K*_*D*_ difficult, the measured association rate constants varied over three orders of magnitude. The 3 mAbs with the highest on-rate constants were custom-made IgG from Precision Antibody (10F5, 10C1, 6C8). Three commercial antibodies had the lowest *k*_*on*_ values, two of which were IgM (MPFM-55A and PTL3). This was not surprising, since IgM generally have low-affinity, high-avidity interactions with their targets [12].

To identify the best-performing antibody pairs for an HRP2 sandwich immunoassay, all 225 possible pairs of anti-HRP2 mAbs (15 × 15 matrix) were screened in a checkerboard format. The average signal-to-noise ratio (S/N) for each pair was determined by dividing the average A_450_ at 34.1 pM of rcHRP2 by the average absorbance of the blank (Fig. S1). Several mAbs, such as MPFG, C1-13, 4D6, 8D3, 11H7, and 12F12 performed poorly as capture, while MPFM, PTL3, 2g6 and 0445 performed well as capture elements. Additionally, numerous custom mAbs were successful as detection components, including 4D6, 6C8, 10C1, 10F5, 11E10, 12D4 and 12F12.

With the quantification of individual mAb-HRP2 interactions by BLI and the relative ranking of anti-HRP2 mAb pairs in a checkerboard ELISA screening comes the question: can BLI be used to predict antibody pair performance in a traditional plate ELISA format?

Fig. 1 relates the measured kinetic parameters of anti-HRP2 capture and detection antibodies to the S/N measured for each pair in the checkerboard ELISA. In these plots, a kinetic parameter for the capture antibody is plotted on the abscissa, and a kinetic parameter for the detection antibody is plotted on the ordinate. The size of the circle at the ordinate pair (capture, detection) represents the relative S/N for that pair measured by ELISA. Larger circles indicate better-performing ELISA antibody pairs, while small circles indicate poor antibody pairs. The best-performing mAb pairs in the ELISA format are associated with capture and detection antibodies with low *K*_*D*_ values, since the largest circles are concentrated in the lower left-hand corner of the plot in Fig. 1A. This result is anticipated, as one would expect stronger interactions to lead to better ELISA performance. However, when the ELISA data is compared to *k*_*on*_ and *k*_*off*_ values of the capture and detection antibodies, interesting trends emerge. Fig. 1B plots *k*_*on*_ values for capture and detection mAbs versus ELISA S/N. In this visualization, it becomes apparent that no trend relates ELISA S/N to *k*_*on*_ of capture antibodies, since the sizes of the circles neither increase nor decrease along the x-axis. However, there is a dependence of ELISA S/N on the *k*_*on*_ of the detection antibody along the y-axis; greater detection antibody *k*_*on*_ results in better ELISA antibody pair performance for anti-HRP2 mAbs. For *k*_*off*_ (Fig. 1C), the trends are less clear, since there were fewer discrete *k*_*off*_ values for the anti-HRP2 mAbs compared to *k*_*on*_ values. However, Fig. 1D shows that the highest concentration of high-performing anti-HRP2 ELISA antibody pairs occurs when capture antibody *k*_*off*_ is low and detection antibody *k*_*on*_ is high.

**Fig. 1:**
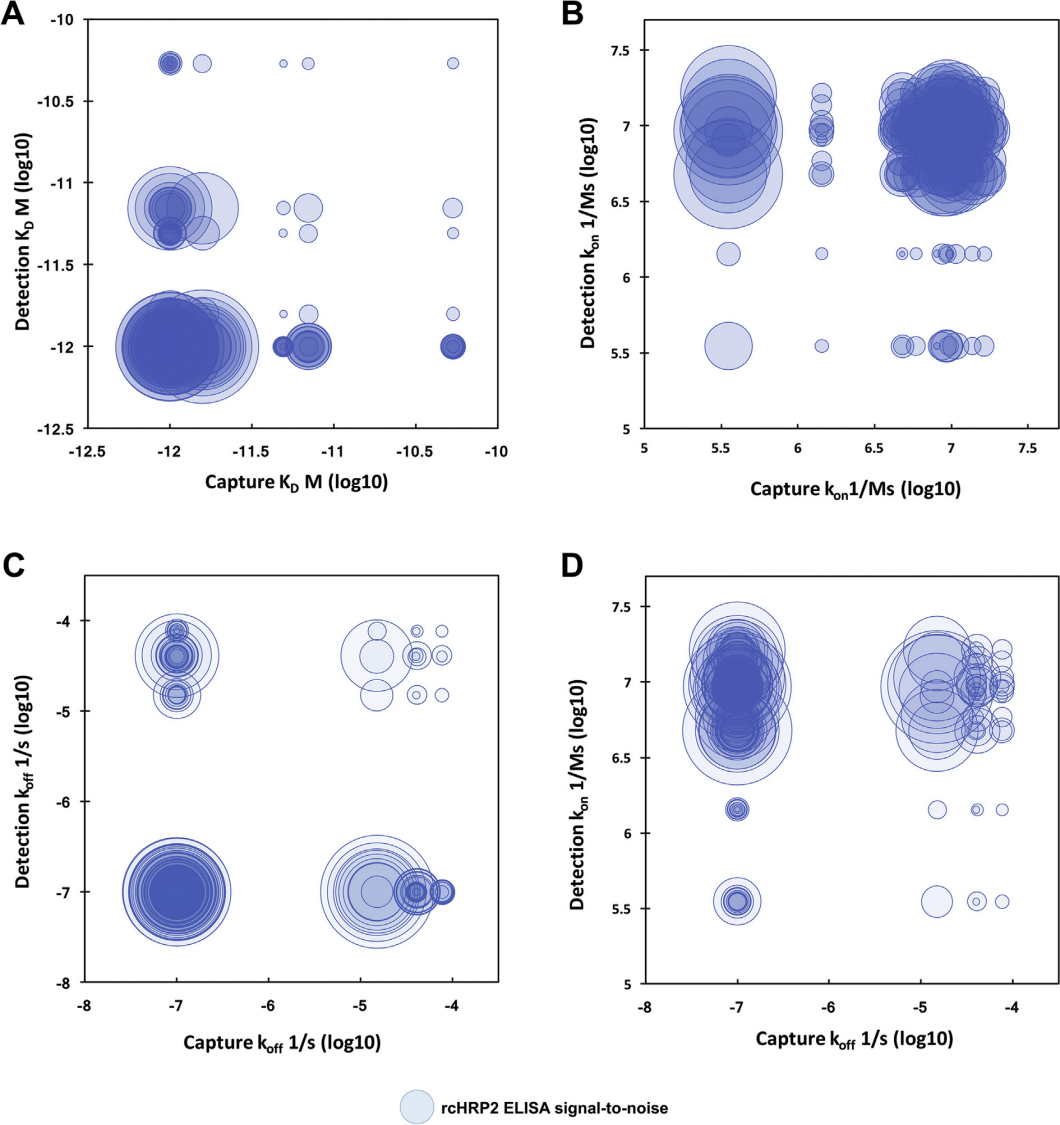
Relationship between anti-HRP2 (A) *K*_*D*_, (B) *k*_*on*_, and (C) *k*_*off*_ in the capture (x-axis) and detection (y-axis) positions as measured by BLI and ELISA signal-to-noise ratios (size of circles). (D) Plot of capture *k*_*off*_ (x-axis) vs. detection *k*_*on*_ (y-axis) vs. ELISA signal-to-noise (circles). Larger circles represent higher S/N. Note: only anti-HRP2 IgG pairs are plotted.

Using these BLI parameters, it would be predicted that pairs with 10F5, 10C1, and 6C8 in the detection position paired with capture mAbs with low off-rate constants, such as the two IgM mAbs (MPFM-55A, PTL-3) or an IgG such as 0445, would perform well in an ELISA format. In fact, these pairs are some of the top-performers in the ELISA checkerboard screening, despite the low *k*_*on*_ values for the capture mAbs (Fig. S1). Capture antibodies with low *k*_*off*_ may be favored due to the high number of washes and additional incubation steps the capture mAb-HRP2 complex is subjected to during the ELISA protocol. In contrast, *k*_*on*_ may be most important for detection antibodies, since the time scale of this interaction is lower. These emperical results agree with a previously reported model [13]. Notably, kinetic parameters were determined by BLI in under 35 min for each mAb, highlighting the advantage of this technique as a rapid screening tool.

These results demonstrate that BLI can be used as a rapid, predictive tool for development of an HRP2 ELISA. Because HRP2 lacks structure and contains a series of repeated epitopes for mAb binding, it is important to ask whether trends observed for HRP2 are generalizable to protein biomarkers with defined tertiary structure and no or few repeat motifs in the sequence. For HRP2, if a capture and detection antibody target the same epitope, both may be able to bind to the protein due to the repeat motifs in the amino acid sequence. However, for targets lacking repeated epitopes, if two antibodies both target the same epitope, it is likely that little-to-no ELISA signal would be observed.

The utility of BLI for quantifying the strength of antibody-antigen interactions is clear, and this work demonstrates that BLI is also useful for predicting the ELISA performance of antibody *pairs* when an antigen contains many repeats in its sequence. There are many targets of interest that could benefit from building a sensitive ELISA from the bottom-up using BLI as a predictive tool. For example, the circulating anodic antigen biomarker for *Schistosoma* infections is decorated with repeating polysaccharide motifs [14], [15]. Further, BLI may be useful for developing ELISAs for viral capsids containing many oligomeric subunits or multimeric proteins with identical subunits.

## Conflict of interest

PATH funded the development of the antibodies and entered into a licensing agreement with A&G to make the antibodies commercially available. PATH and its affiliated authors do not have any financial interest in A&G nor receive any royalties under the license agreement.
