# Supplementary material for: Biolayer interferometry predicts ELISA performance of monoclonal antibody pairs for Plasmodium falciparum histidine-rich protein 2
Source: Anal Biochem. 2017 Oct 1;534:10–3. doi: 10.1016/j.ab.2017.07.010 (PMC5552614; doi:10.1016/j.ab.2017.07.010)
Supplement: Supplemental Information [file mmc1.docx]

**Biolayer interferometry predicts ELISA performance of monoclonal antibody pairs for *Plasmodium falciparum* histidine-rich protein 2**

Markwalter, C. F.;^1^ Jang, I. K.;^2^ Burton, R. A.;^2,3^ Domingo, G. J.;^2^ Wright, D. W.^1^*

^1^ Department of Chemistry, Vanderbilt University, Nashville, TN 37235, USA

^2^ PATH, Seattle, WA, 98121, USA

^3^ Present Address: Center for Genomic Interpretation, Sandy, UT, USA

*Email address: david.wright@vanderbilt.edu

Table of Contents:

| Materials and Methods.............................................................................................................. | S-2 |
| --- | --- |
| Anti-HRP2 monoclonal antibodies assessed by BLI and ELISA............................................. | S-6 |
| Biolayer interferometry binding profiles for anti-HRP2 IgG................................................... | S-7 |
| Biolayer interferometry binding profiles for anti-HRP2 IgM.................................................. | S-10 |
| Kinetic parameters for anti-HRP2 IgG and IgM...................................................................... | S-11 |
| S/N for checkerboard ELISA of 225 anti-HRP2 mAb pairs.................................................... | S-12 |

**Materials and Methods**

*Materials*

Recombinant ITG histidine-rich protein 2 (rcHRP2) and HRU20 were kindly provided by D. Sullivan (Johns Hopkins University, Baltimore, MD). Protein concentrations were determined in triplicate by amino acid analysis (University of Nebraska, Protein Structure Core Facility, Omaha, NE). *In vitro* *P. falciparum* culture supernatant was used as source of native HRP2 protein for monoclonal antibody (mAb) screening. Sources for commercial anti-HRP2 antibodies are listed in Tables S1 and S2.

*Antibody Production*

The custom anti-HRP2 mAbs were developed under contract with Precision antibody, Inc (Columbia, MD). For mice immunization, recombinant HRP2 was mixed with proprietary adjuvant and immunized into three BALB/c mice according to proprietary immunization protocols. Tail bleeds after administration of a final dose were analyzed for anti-HRP2 titers by direct ELISA. When a desirable antibody titer (A_450_ > 2.0 at 1:100,000 dilution) was achieved, a mouse was euthanized and its spleen removed under aseptic conditions. A single-cell suspension of splenocytes was prepared, and cells were subjected to electrofusion with myeloma cells. Cells were distributed into 96-well plates, and incubated at 37°C. The hybridoma culture supernatants were then screened for the presence of anti-HRP2 mAbs by direct ELISA. The direct ELISA was performed on microtiter plates, and all reagents were diluted in ELISA buffer (PBS containing 5% milk). Plates were coated with HRP2 ITG or HRU20 at two protein concentrations, 100 ng/well or 1 ng/well in ELISA buffer, and washed with washing buffer (PBS containing 0.05% Tween 20). Hybridoma growth media diluted (1:2) in PBS containing 5% milk was used as negative control. Calf serum diluted (1:1,000) in PBS containing 5% milk was used as positive control.

The mAbs were purified by affinity chromatography using protein G from hybridoma culture supernatant followed by acid elution and neutralization. The Abs were buffer exchanged into PBS and tested for purity with SDS-PAGE. The concentration of purified mAbs was determined by absorbance at 280 nm.

*Determination of kinetic parameters of mAbs with BLI.*

All IgG antibodies were biotinylated in PBS with EZ-Link NHS-PEG4-Biotin (ThermoFisher #21329) at 20x molar excess according to the commercial protocol. Unreacted biotin was removed using Thermo Zeba Spin Columns (ThermoFisher # 89882). Kinetics experiments were performed using an OctetRed96 system equipped with streptavidin biosensors (ForteBio LLC, Fremont, CA). All solutions were made in octet kinetics buffer (1x PBS with 0.1% BSA and 0.02% Tween-20). Each kinetic experiment consisted of 5 steps: (1) streptavidin biosensors were equilibrated in kinetics buffer for 3 to 5 minutes, (2) a biotinylated mAb (0.5 µg/ml) was loaded onto the streptavidin biosensors for 400 seconds (3) a baseline was established in kinetics buffer for 1 minute, (4) rcHRP2 antigen (0 - 14 nM) in kinetics buffer was associated to the functionalized sensors for 400 seconds, and (5) sensors were placed in kinetics buffer, and the antigen was allowed to dissociate for 15 minutes. The assay was performed at 26°C with 1000 rpm plate rotation. Software provided with the Octet system (version 7.1) was used to fit the data to a one-to-one model and obtain *k_on_*, *k_off_*, and *K_D_* values.

To determine the kinetic parameters of anti-HRP2 IgM, rc*Pf*HRP2 was biotinylated at 20x molar excess as noted above. Kinetics experiments were performed with streptavidin biosensors. In these experiments, 10 nM rcHRP2 was loaded onto the tips, and the IgM antibodies were allowed to associate and dissociate from the rcHRP2-functionalized sensors. Loading, association, and dissociation times were optimized for each IgM antibody, and are shown below.

| Step/IgM | MPFM-55A | PTL3 |
| --- | --- | --- |
| Loading | 500 s | 150 s |
| Association | 400 s | 700 s |
| Dissociation | 900 s | 900s |

*Screening antibody pairs by ELISA.*

Antibodies were conjugated to horseradish peroxidase using EZ-Link Plus Activated Peroxidase kit (Thermo Scientific #31489). Briefly, 0.2 mg mAb in 100 μl of carbonate bicarbonate buffer was incubated with 0.2 mg of horseradish peroxidase in 200 µl of ultrapure water for 1 hour at room temperature. Next, 4 μl of sodium cyanoborohydride was added and incubated for 15 minutes at room temperature. The reaction was stopped by adding 4 μl of quenching buffer and incubating for 15 minutes. The reaction was then subjected to desalting using Vivaspin 500 (Sartorius # VS0101).

A checkerboard-formatted sandwich ELISA was performed with all possible combinations of 9 custom and 6 commercial HRP2-specific antibodies (Table S1) to determine the best antibody pairs for HRP2 detection according to a modified method [18]. Briefly, a 96-well plate (Costar, #3361) was coated with 100 μl/well of each antibody solution at 1 μg/ml in PBS. The plates were sealed and incubated overnight at 4°C. The antibody solutions were discarded, and the plate was blocked for 2 hours with 200 μl/well of 2 % bovine serum albumin (BSA) in PBS. The plates were washed five times with 200 μl/well PBS, pH 7.4, with 0.05% (PBST). Next, 100 μl of rcHRP2 (1 ng/ml; 34.1 pM) in PBST was placed in the wells, and the plates were incubated in a humidified chamber for 1 hour at room temperature. A pair of commercial antibodies (Immunology Consultant laboratory, INC), MPFM- 55A and MPFG-55A, previously described for detecting HRP2, was used as a positive control [18]. Next, the plate was washed five times before 100 µl of 0.5 µg/ml of the detection antibody-HRP conjugate in 2% bovine serum albumin and 0.1 % Tween 20 in PBS was added to each well. The plate was incubated for one hour at room temperature and washed five times with PBST. The enzymatic reaction was visualized using TMB substrate with hydrogen peroxide (Sigma, T0440) and stopped with 50 μl of 1 M H_2_SO_4_. Spectrophotometric analysis was performed at 450 nm using SpectraMAX 340 Microplate spectrophotometer (Molecular Devices, Sunnyvale, CA).

**Table S1.** Anti-HRP2 monoclonal antibodies assessed by BLI and ELISA.

| **Source** | **Clone** | **Isotype** |
| --- | --- | --- |
| ICL | MPFG-55A | IgG |
|  | MPFM-55A | IgM |
| NBI | C1-13 | IgG |
|  | PTL-3 | IgM |
| Vista Diagnostics | 2g6 | IgG |
|  | 0445 | IgG |
| Precision Antibody | 4D6 | IgG |
|  | 6C8 | IgG |
|  | 8D3 | IgG |
|  | 10C1 | IgG |
|  | 10F5 | IgG |
|  | 11E10 | IgG |
|  | 11H7 | IgG |
|  | 12D4 | IgG |
|  | 12F12 | IgG |

**Table S2**. Binding profiles and fit residuals for anti-HRP2 IgG. Data is shown in black, while calculated fits are shown in red.

| **IgG Clone** | **Binding profile** | **Residual plot** |
| --- | --- | --- |
| **MPFG-55A** | 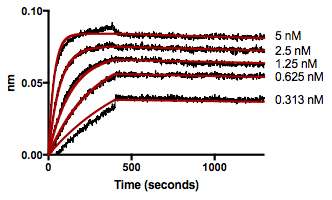 | 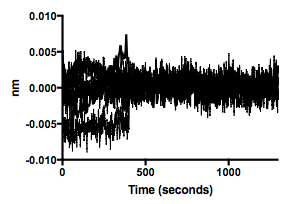 |
| **C1-13** | 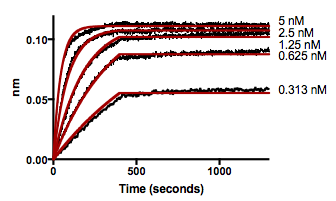 | 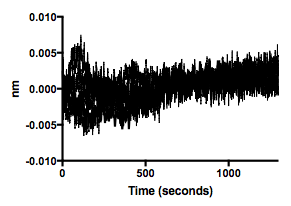 |
| **0445** | 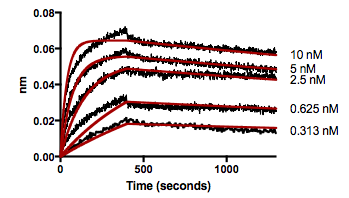 | 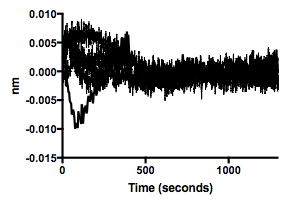 |
| **2g6** | 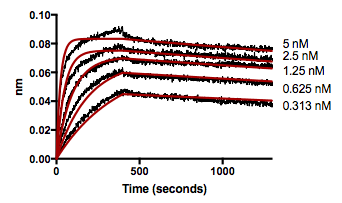 | 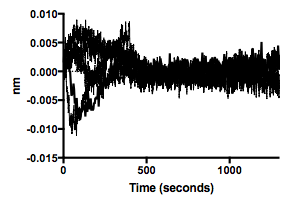 |
| **4D6** | 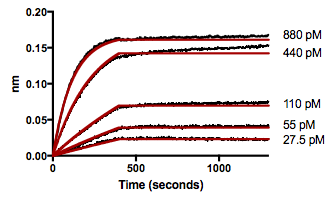 | 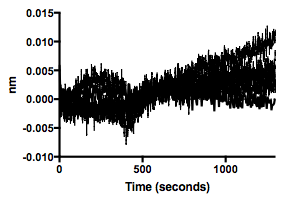 |
| **6C8** | 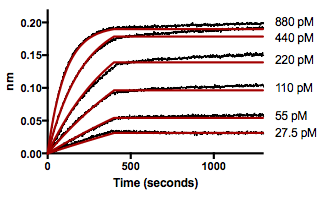 | 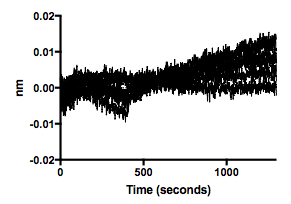 |
| **8D3** | 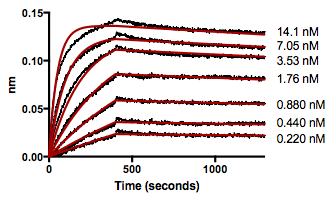 | 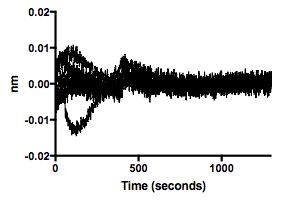 |
| **10C1** | 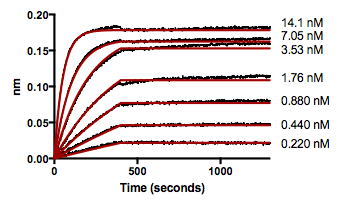 | 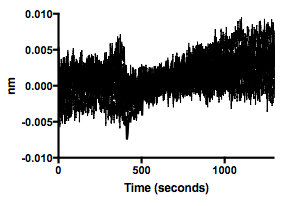 |
| **10F5** | 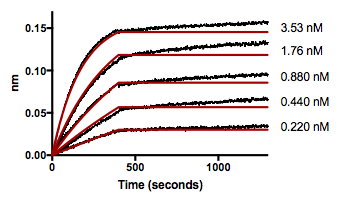 | 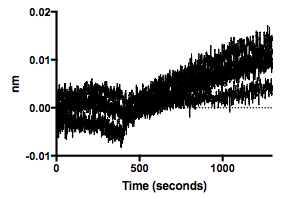 |
| **11E10** | 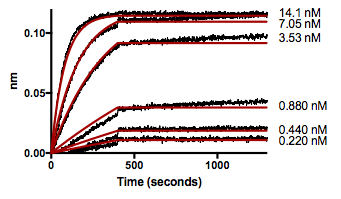 | 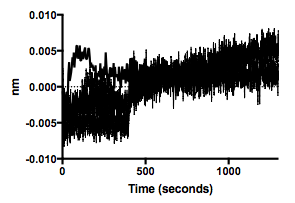 |
| **11H7** | 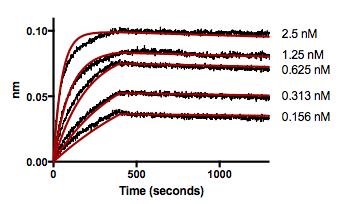 | 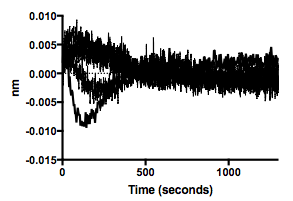 |
| **12D4** | 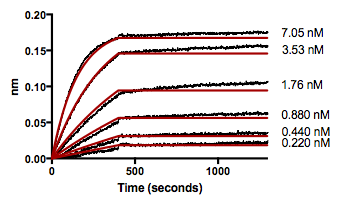 | 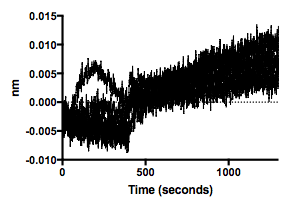 |
| **12F12** | 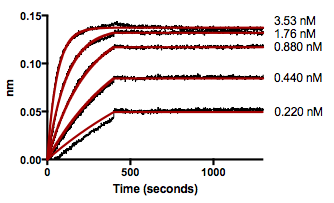 | 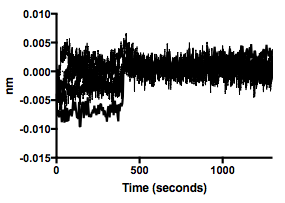 |

**Table S3**. Binding profiles and fit residuals for anti-HRP2 IgM

| **IgM Clone** | **Binding profile** | **Residual plot** |
| --- | --- | --- |
| **MPFM-55A** | 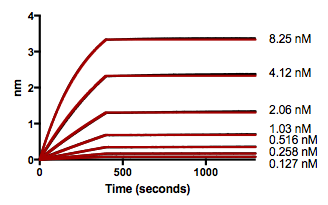 | 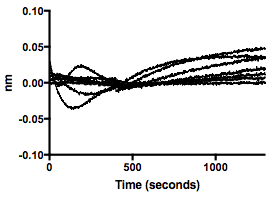 |
| **PTL3** | 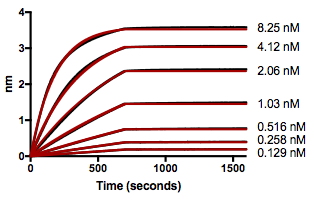 | 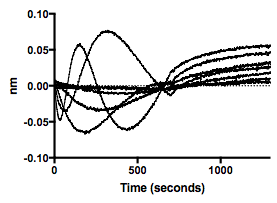 |

**Table S4**. Kinetic parameters for anti-HRP2 antibodies measured by BLI.

| Source | Clone | Class | Kinetic parameter | | |
| --- | --- | --- | --- | --- | --- |
|  |  |  | *K_D_* M x 10^-12^ | *k_on_* 1/Ms x 10^5^ | *k_off_* 1/s x 10^-7^ |
| ICL | MPFG-55A | IgG | 7 ± 1 | 58.8 ± 0.3 | 410 ± 10 |
|  | MPFM-55A | IgM | < 1.0 | 4.22 ± 0.01 | < 1.0 |
| NBI | C1-13 | IgG | < 1.0 | 47.7 ± 0.5 | < 1.0 |
|  | PTL-3 | IgM | < 1.0 | 6.70 ± 0.01 | < 1.0 |
| Vista Diagnostics | 2g6 | IgG | 1.6 ± 0.1 | 93.4 ± 0.6 | 150 ± 15 |
|  | 0445 | IgG | < 1.0 | 3.52 ± 0.02 | < 1.0 |
| Precision Antibody | 4D6 | IgG | < 1.0 | 96.9 ± 0.5 | < 1.0 |
|  | 6C8 | IgG | < 1.0 | 107.0 ± 0.7 | < 1.0 |
|  | 8D3 | IgG | 53.5 ± 0.1 | 14.30 ± 0.06 | 766 ± 12 |
|  | 10C1 | IgG | < 1.0 | 137.0 ± 0.4 | < 1.0 |
|  | 10F5 | IgG | < 1.0 | 164.0 ± 0.7 | < 1.0 |
|  | 11E10 | IgG | < 1.0 | 87.4 ± 0.5 | < 1.0 |
|  | 11H7 | IgG | 4.9 ± 0.1 | 80.5 ± 0.4 | 400 ± 20 |
|  | 12D4 | IgG | < 1.0 | 93.0 ± 0.8 | < 1.0 |
|  | 12F12 | IgG | <1.0 | 47.8 ± 0.2 | < 1.0 |

**Figure S1**. ELISA signal-to-noise ratios for checkerboard screening of anti-HRP2 mAb pairs

**
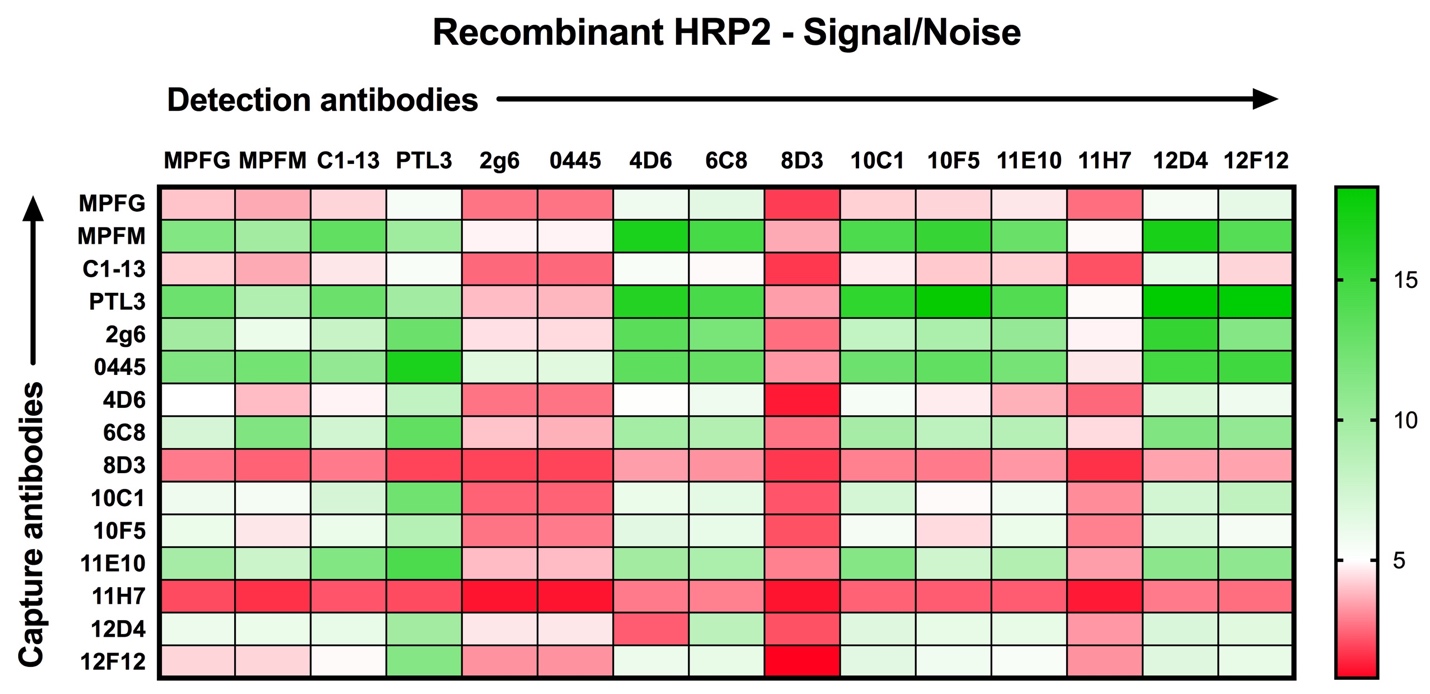
**
